# Adventitious agents and vaccines.

**DOI:** 10.3201/eid0707.017735

**Published:** 2001

**Authors:** P R Krause

**Affiliations:** Food and Drug Administration, Bethesda, Maryland 20892, USA. krause@cber.fda.gov


### Adventitious Agents and Vaccines

**Philip R. Krause**
Food and Drug Administration, Bethesda, Maryland, USA

A discussion of adventitious agents and vaccines is pertinent in the context of emerging infectious diseases. Many novel vaccines are produced in animal cell substrates, and emerging infectious diseases may theoretically be transmitted from animals to humans through these vaccines. The challenge of identifying potential adventitious agents in vaccines closely parallels the challenge of identifying the agents causing particular emerging infectious diseases. Thus, the major focus of this discussion will be the approaches used in a regulatory setting to ensure that vaccines are devoid of adventitious agents.

Maximal vaccine benefit is achieved when vaccination rates are sufficiently high to achieve herd immunity. Because vaccines are administered to healthy children, it is especially important that parents, pediatricians, and the public at large feel confident that the vaccines are safe. Knowing that vaccines are free from adventitious agents is a large component of this confidence. Thus, ensuring that vaccine products that are administered to the public do not contain adventitious agents is a regulatory goal. Of course, the potential for the presence of adventitious agents in any vaccine must also be evaluated in terms of the overall benefit of the product.

In the past, biologic products have served as vectors for viral diseases. Examples include the contamination of yellow fever vaccine with hepatitis B virus in the 1940s (because a human-derived excipient contained hepatitis B virus), contamination of early polio and adenovirus vaccines with simian virus 40 in the late 1950s and early 1960s, contamination of blood products with hepatitis viruses and HIV, and contamination of dura mater grafts with the Creutzfeldt-Jakob disease agent. In these examples, either human or animal materials used in production usually caused the contamination.

Production of viral vaccines generally involves inoculation of a cell substrate with a vaccine seed and purification of bulk product from these cells after a sufficient time for replication of the virus or production of vaccine proteins. Other raw materials (e.g., tissue culture reagents, stabilizers) may be added to the product at various stages of production. Thus, adventitious agents could theoretically enter a viral vaccine through any of these ingredients. Close control of the vaccine manufacturing environment (by producing vaccines in sophisticated modern facilities), appropriate testing of the raw materials, and testing of both the bulk and final products can help ensure that adventitious agents have not entered the vaccine. Most vaccines are subjected to inactivation or purification steps that can reduce the likelihood of contamination with adventitious agents.

Current research on emerging infectious diseases may help provide further assurance that new vaccines do not contain adventitious agents. Powerful methods used to discover viruses associated with emerging infectious diseases are also being adapted to ensure that new vaccines (some of which may be produced in novel cell substrates) are free of adventitious agents.

Address for correspondence: Philip R. Krause, Food and Drug Administration, Building 29A, Room 1C16, HRM-457, 29 Lincoln Drive, Bethesda, MD 20892, USA; fax: 301-496-1810; e-mail: krause@cber.fda.gov

